# Association of Glucagon-like Peptide-1 Receptor Agonist Use with Stroke and Mortality Outcomes in Asymptomatic Intracranial Atherosclerotic Disease: Propensity Score-Matched Real-World Analysis

**DOI:** 10.3390/neurolint18050098

**Published:** 2026-05-21

**Authors:** Pranjal Rai, Daniel Mandel, Girish Bathla, Vidhi Dhaduk, Radhika Rajeev, Jay Kakadiya, Huanwen Alvin Chen, Hamza A. Salim, Ahmed Y. Azzam, Muhammed Amir Essibayi, Brian Connolly, Marc Buzzelli, Vivek S. Yedavalli, Majid Khan, Adam A. Dmytriw, David J. Altschul, Matthew K. McIntyre, Marco Colasurdo, Ajay Malhotra, Dheeraj Gandhi, Dhairya A. Lakhani

**Affiliations:** 1 Department of Radiology, Mayo Clinic, 200 1st Street Southwest, Rochester, MN 55902, USA; bathla.girish@mayo.edu; 2 Department of Neurology, Rockefeller Neuroscience Institute, West Virginia University, Morgantown, WV 26506, USA; 3 Shantabaa Medical College and General Hospital, Amreli 365601, Gujarat, India; vidhidhaduk1@gmail.com; 4 Department of Radiology, Massachusetts General Hospital, Harvard University, Boston, MA 02114, USA; drradhikarajeev9@gmail.com; 5 Government Medical College, Surat 395001, Gujarat, India; jaykakadiya07@gmail.com; 6 Department of Neurosurgery, University of Maryland Medical Center, Baltimore, MD 21201, USA; hwchen@som.umaryland.edu (H.A.C.); dheeraj.gandhi@som.umaryland.edu (D.G.); 7 Department of Neuroradiology, MD Anderson Medical Center, Houston, TX 77030, USA; hamzasleeem@gmail.com; 8 Department of Neuroradiology, Rockefeller Neuroscience Institute, West Virginia University, Morgantown, WV 26506, USA; ahmedyazzam@gmail.com (A.Y.A.); bdconnolly@hsc.wvu.edu (B.C.); marc.buzzelli@hsc.wvu.edu (M.B.); dhairya.lakhani@hsc.wvu.edu (D.A.L.); 9 Department of Neurological Surgery and Montefiore-Einstein Cerebrovascular Research Lab, Montefiore Medical Center, Albert Einstein College of Medicine, Bronx, NY 10461, USA; mamiressibayi@gmail.com (M.A.E.); daltschu@montefiore.org (D.J.A.); 10 Department of Radiology and Radiological Sciences, Johns Hopkins Medical Center, Baltimore, MD 21205, USA; vyedava1@jhmi.edu (V.S.Y.); mkhan9@jhmi.edu (M.K.); 11 Nuffield Department of Surgical Sciences, Medical Sciences Division, University of Oxford, Oxford OX1 1NF, UK; adamdmytriw@gmail.com; 12 Neuroendovascular Program, Massachusetts General Hospital, Harvard University, Boston, MA 02114, USA; 13 Neurointerventional & Neuroanalytics Consortium (NAN-C), School of Medicine, Toronto Metropolitan University, Toronto, ON M5B 2K3, Canada; 14 Department of Neurosurgery, Oregon Health & Science University, Portland, OR 97239, USA; mcintyma@ohsu.edu; 15 Department of Interventional Radiology, Oregon Health & Science University, Portland, OR 97239, USA; mcolasurdo@gmail.com; 16 Department of Radiology, Yale New Haven Hospital, New Haven, CT 06510, USA; ajay.malhotra@yale.edu

**Keywords:** intracranial atherosclerosis, stroke, mortality, semaglutide, glucagon-like peptide 1 agonists, propensity matching

## Abstract

Background: Asymptomatic intracranial atherosclerotic arterial stenosis (ICAS) is an underrecognized entity for which vascular risk-factor optimization is the primary management strategy, with no current indication for routine antiplatelet therapy or endovascular intervention for primary stroke prevention. Glucagon-like peptide-1 receptor agonists (GLP-1RAs) reduce major adverse cardiovascular events, including stroke, in high-risk cardiometabolic populations, but their association with outcomes in asymptomatic ICAS is yet to be evaluated. The present study aims to evaluate the association between GLP-1RA use and cerebrovascular outcomes in adults with asymptomatic ICAS. Materials and Methods: We used the TriNetX US Collaborative Network (71 healthcare organizations) to identify adults (≥18 years) with ICAS between 1 January 2016 and 31 December 2025, and excluded patients with prior cerebral infarction, intracranial hemorrhage, or cerebrovascular ischemic syndromes. Exposure was defined as initiation of any GLP-1 receptor agonist (lixisenatide, semaglutide, liraglutide, tirzepatide, dulaglutide) during the 6 months before or on the date of index ICAS diagnosis. Outcomes were assessed at 1 year, and included ischemic stroke, all-cause mortality, and a composite of ischemic stroke or mortality. Propensity-score matching (1:1) was performed, including demographics, vascular risk factors, comorbidities, antithrombotics, lipid/diabetes therapies, and cardiometabolic laboratory/physiologic measures. Results: Before matching, 1746 GLP-1RA users and 71,792 non-users met inclusion criteria; after matching, 1728 patients remained in each cohort. GLP-1RA use was associated with lower 1-year risk of ischemic stroke (4.40% vs. 6.10%; hazard ratio [HR] 0.70, 95% CI 0.52–0.95; *p* = 0.044), lower all-cause mortality (3.40% vs. 9.40%; HR 0.35, 95% CI 0.26–0.47; *p* < 0.001), and lower composite outcome risk (7.50% vs. 15.00%; HR 0.48, 95% CI 0.39–0.59; *p* < 0.001). Notably, these associations were observed despite matching for HbA1c, LDL cholesterol, BMI, and systolic blood pressure, suggesting potential effects beyond measured cardiometabolic risk profiles. Conclusions: In this large, propensity-matched cohort of adults with a-ICAS, GLP-1RA use was associated with lower ischemic stroke, all-cause mortality, and composite outcome at 1 year. These findings are hypothesis-generating and require further prospective studies to confirm this observation.

## 1. Introduction

Intracranial atherosclerotic arterial stenosis (ICAS) is characterized by the accumulation of cholesterol, fibrous tissue, and inflammatory cells within the walls of the major cerebral vasculature and is biologically and clinically distinct from extracranial carotid atherosclerosis [1,2]. ICAS is among the most common causes of ischemic stroke worldwide, accounting for approximately 10–16% of ischemic strokes in White populations in Europe and the United States, and 30–50% in many Asian populations, with a high burden also reported among Black and Hispanic populations [3]. In the general US population, intracranial plaque is common (approximately 34%) and clinically meaningful stenosis (defined as ≥50% luminal narrowing) is present in roughly 9% patients, with some studies also reporting higher prevalence in men than women [4].

Clinically, the distinction between asymptomatic and symptomatic ICAS is important. Asymptomatic ICAS is generally detected incidentally on CT or MR angiograms, or MRI vessel wall imaging performed for unrelated reasons (e.g., headache, dizziness, trauma, cognitive symptoms, or other neurologic complaints) [5,6,7,8]. It is associated with a relatively low annual stroke risk (~0.6–2.8%) compared to symptomatic ICAS, which follows a more malignant course with recurrent stroke rates reported as high as ~12–20% at 1 year despite standard therapy [3,9,10]. The absence of a qualifying ischemic event does not necessarily imply biological quiescence, rather asymptomatic ICAS may represent an earlier or clinically silent phase of intracranial atherosclerosis, particularly in patients with substantial cardiometabolic risk burden.

Accordingly, while contemporary management guidelines largely focus on secondary prevention in symptomatic ICAS, evidence-based strategies for asymptomatic ICAS remain limited. In clinical practice, management is generally extrapolated from global vascular risk reduction, emphasizing intensive control of weight, diabetes, blood pressure, smoking status, lipids and other vascular risk factors, rather than ICAS-specific, targeted therapies [11,12]. However, asymptomatic ICAS occupies a clinically ambiguous space: it is often discovered incidentally, yet may also serve as a marker of heightened systemic and cerebrovascular risk. This creates a need to better understand whether cardiometabolic therapies associated with vascular benefit in other high-risk populations are also associated with improved outcomes in patients with asymptomatic ICAS.

Glucagon-like peptide-1 receptor agonists (GLP-1RAs), initially developed for type 2 diabetes to enhance insulin secretion and suppress glucagon, have rapidly emerged as cardiometabolic agents with reproducible benefits on major adverse cardiovascular events, including stroke, in high-risk populations [13,14]. Meta-analyses of randomized trials in patients with type 2 diabetes suggest that GLP-1RAs reduce stroke risk by approximately 15–17%, an effect hypothesized to reflect pleiotropic mechanisms extending beyond glycemic control, including anti-atherosclerotic actions, potential neuroprotective effects (central anti-inflammatory and antioxidative pathways), and favorable changes in weight, blood pressure, and lipid profiles [15,16]. Collectively, these observations motivate evaluation of GLP-1RAs in asymptomatic ICAS, a population in which risk-factor optimization is central but targeted cerebrovascular preventive strategies remain incompletely defined.

Despite increasing use of GLP-1RAs and growing evidence for cardiovascular benefit, their association with outcomes in patients with asymptomatic ICAS has not been well characterized. This is an important gap because asymptomatic ICAS differs from extracranial carotid atherosclerosis and generalized cardiovascular disease in anatomy, mechanisms of ischemia, available preventive strategies, and clinical decision-making. Furthermore, randomized trials of GLP-1RAs have generally not been designed around imaging-defined intracranial atherosclerosis, stenosis severity, or cerebrovascular phenotypes. Real-world EHR data can therefore provide an initial signal in a population that is difficult to assemble prospectively, while recognizing that such analyses remain vulnerable to residual confounding, exposure misclassification, and coding limitations. Accordingly, we used a large multicenter EHR network to evaluate whether GLP-1RA use was associated with 1-year ischemic stroke, all-cause mortality, and a composite of ischemic stroke or mortality among adults with coded asymptomatic ICAS.

## 2. Materials and Methods

### 2.1. Study Design

This retrospective study leveraged the TriNetX US Collaborative Network, a federated electronic health record network that provides access to de-identified clinical data from participating health care organizations in the United States. The network includes structured data on diagnoses, procedures, medications, laboratory values, and demographic information. At the time of analysis, the US Collaborative Network included 71 health care organizations, all of which were queried and responded to the cohort definitions. Because TriNetX provides only de-identified data, this study was exempt from institutional review board review and informed consent requirements. Study cohorts, exposure definitions, covariates, and outcomes were identified using structured diagnosis and medication concepts, including International Classification of Diseases, Tenth Revision (ICD-10) coding, as detailed in Supplementary Table S1.

### 2.2. Participants

Adults aged 18 years or older with a diagnosis code for ICAS were identified between 1 January 2016 and 31 December 2025. To operationalize an asymptomatic ICAS cohort, we excluded patients with cerebral infarction, nontraumatic intracerebral hemorrhage, other nontraumatic intracranial hemorrhage, transient cerebral ischemic attacks and related syndromes, and vascular syndromes of the brain in cerebrovascular diseases, prior to and on the day of ICAS diagnosis, as defined by the ICD-10 codes listed in Supplementary Table S1. These exclusions were intended to reduce inclusion of patients with symptomatic ICAS at baseline. However, because symptom status, imaging findings, and adjudicated neurologic presentations were not available in structured form, this cohort should be interpreted as patients with coded asymptomatic ICAS rather than centrally adjudicated imaging-confirmed asymptomatic ICAS.

### 2.3. Exposure and Outcomes

The primary exposure was GLP-1RA use documented during the 6 months before or on the date of asymptomatic ICAS diagnosis. Two cohorts were established: (1) patients with asymptomatic ICAS diagnosis who received GLP-1RA—dulaglutide, liraglutide, semaglutide, tirzepatide, or lixisenatide—within 6 months exposure period (GLP-1RA group), and (2) patients with asymptomatic ICAS diagnosis without any GLP-1RA exposure (non-GLP-1RA group). GLP-1RAs were analyzed as a medication class; stratified analyses by individual agent, dose, duration, titration schedule, adherence, persistence, or discontinuation were not performed. Patients were evaluated over a period of 1 year (365 days) after the index ICAS diagnosis. The index date for both cohorts was the date of asymptomatic ICAS diagnosis, and follow-up began after the index diagnosis date. This exposure definition was selected to ensure that exposure status was established before outcome follow-up began and to reduce immortal time bias.

Outcomes included ischemic stroke, all-cause mortality, and a composite of ischemic stroke or all-cause mortality. Ischemic stroke was defined using the ICD-10-CM code for cerebral infarction. Mortality was identified using the TriNetX deceased demographic variable. The composite outcome was defined as the occurrence of either ischemic stroke or death during the follow-up window. In the TriNetX survival analysis, censoring was applied after the last recorded fact in a patient’s electronic health record. Kaplan–Meier survival analyses, measures of association, and outcome counts were generated within the TriNetX platform.

### 2.4. Statistical Analysis

Propensity score matching (PSM) in a 1:1 ratio was performed within TriNetX to reduce measured baseline differences between GLP-1RA users and non-users. Matching included 50 baseline characteristics across demographic, diagnostic, medication, laboratory, and physiologic domains. Demographic variables included age at index, sex, White race, and Black or African American race. Diagnostic covariates included atrial fibrillation and flutter, hypertension, hyperlipidemia, diabetes mellitus, heart failure, arterial fibromuscular dysplasia, alcohol-related disorders, liver disease, chronic kidney disease, coagulation defects and other hemorrhagic conditions, peripheral vascular disease, ischemic heart disease, headache, psychoactive substance use disorders, history of nicotine dependence, and stenosis of precerebral arteries (internal carotid arteries) not resulting in cerebral infarction.

Medication covariates included anticoagulants, antiplatelet agents, lipid-lowering therapies, antihypertensive medications, and non-GLP-1RA glucose-lowering medications. Specifically, these included warfarin, rivaroxaban, apixaban, dabigatran, aspirin, ticagrelor, clopidogrel, evolocumab, alirocumab, inclisiran, atorvastatin, rosuvastatin, pravastatin, simvastatin, ezetimibe, metformin, empagliflozin, dapagliflozin, glipizide, sitagliptin, glimepiride, beta blockers, diuretics, calcium channel blockers, angiotensin II receptor blockers, and angiotensin-converting enzyme inhibitors. Laboratory and physiologic variables included LDL cholesterol, hemoglobin A1c, body mass index, and systolic blood pressure.

Covariate balance was assessed using standardized mean differences. An absolute standardized mean difference less than 0.12 was considered acceptable balance. Baseline characteristics were summarized as frequencies with percentages for categorical variables and as means with standard deviations for continuous variables, with comparisons performed using chi-square (or Fisher’s exact) test for categorical variables and *t*-tests for continuous variables. Associations between GLP-1RA exposure and 1-year outcomes were assessed using TriNetX Compare Outcomes analyses. Measures of association included risk ratios, risk differences, and odds ratios, each with corresponding 95% confidence intervals (CI). Time-to-event analyses employed Kaplan–Meier methods, with between-group comparisons assessed using log-rank testing. Cox proportional hazards models were used to estimate hazard ratios with 95% CIs to estimate relative effects. Patients were censored after the last recorded fact in their electronic health record. In accordance with TriNetX governance requirements, outcomes involving fewer than 10 events were reported as “≤10.” Statistical tests were two-sided, with *p* < 0.05 indicating statistical significance. All analyses were performed within the TriNetX platform.

## 3. Results

Before PSM, 1746 patients were included in the GLP-1RA cohort and 71,792 patients were included in the non-GLP-1RA cohort. After 1:1 PSM, 1728 remained in each cohort. After PSM, the mean follow-up was similar but slightly longer for the GLP-1RA cohort as compared to the non-GLP-1RA cohort (287.54 ± 122.71 days vs. 285.22 ± 131.01 days respectively). Supplementary Table S2 summarizes the demographic and comorbidity variables before and after PSM. After matching, most demographic, diagnostic, and medication covariates were well balanced between cohorts. The matched cohorts had similar mean age, sex distribution, race distribution, prevalence of hypertension, hyperlipidemia, atrial fibrillation, heart failure, chronic kidney disease, ischemic heart disease, antiplatelet use, anticoagulant use, statin use, and antihypertensive medication use. However, residual imbalance persisted for selected cardiometabolic variables, including HbA1c and BMI, both of which remained higher in the GLP-1RA cohort after matching. In the TriNetX output, post-matching HbA1c was 7.6 ± 1.9 in the GLP-1RA cohort vs. 7.2 ± 1.8 in the non-GLP-1RA cohort, and BMI was 33.4 ± 7.2 kg/m^2^ vs. 32.2 ± 7.0 kg/m^2^, respectively.

### 3.1. One-Year Ischemic Stroke

At 1 year, ischemic stroke occurred in 76 of 1728 patients in the GLP-1RA cohort and 105 of 1728 patients in the non-GLP-1RA cohort, corresponding to crude event proportions of 4.40% and 6.10%, respectively. The Kaplan–Meier estimated cumulative event probability was also lower in the GLP-1RA cohort than in the non-GLP-1RA cohort: 5.18% vs. 6.98%. GLP-1RA use was associated with a lower hazard of ischemic stroke during 1-year follow-up: HR, 0.70; 95% CI: 0.52–0.95; *p* = 0.044.

### 3.2. One-Year All-Cause Mortality

All-cause mortality occurred in 58 of 1728 patients in the GLP-1RA cohort and 162 of 1728 patients in the non-GLP-1RA cohort, corresponding to crude event proportions of 3.40% and 9.40%, respectively. The Kaplan–Meier estimated cumulative event probability was 4.13% in the GLP-1RA cohort and 10.52% in the non-GLP-1RA cohort. GLP-1RA use was associated with a lower hazard of all-cause mortality: HR, 0.35; 95% CI: 0.26–0.47; *p* < 0.001.

### 3.3. Composite Outcome: Ischemic Stroke or Mortality

The composite outcome of ischemic stroke or all-cause mortality occurred in 129 of 1728 patients in the GLP-1RA cohort and 260 of 1728 patients in the non-GLP-1RA cohort, corresponding to crude event proportions of 7.50% and 15.00%, respectively. The Kaplan–Meier estimated cumulative event probability was 8.90% in the GLP-1RA cohort and 16.75% in the non-GLP-1RA cohort. GLP-1RA use was associated with a lower hazard of the composite outcome: HR, 0.48; 95% CI: 0.39–0.59; *p* < 0.001.

**Figure 1:**
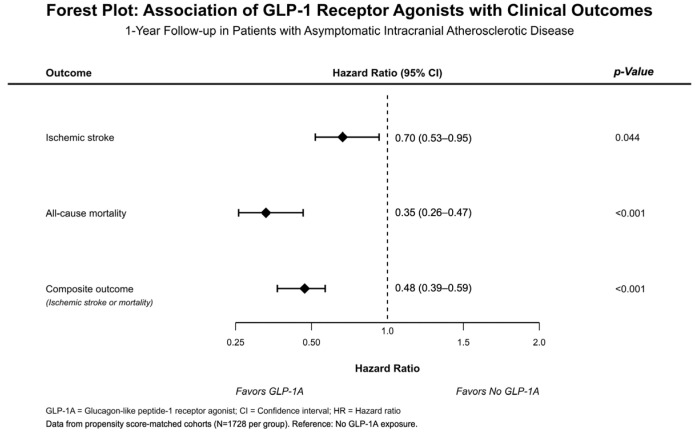
Forest plot of adjusted hazard ratios for clinical outcomes in patients with GLP-1RA use over a 1-year period.

Figure 1 gives a summary of the HRs across all 1-year outcomes. Table 1 summarizes the event counts, Kaplan–Meier estimated event probabilities, hazard ratios, 95% confidence intervals, and *p* values for each outcome within our captured population with and without GLP-1RA exposure.

## 4. Discussion

### 4.1. Principal Findings

In this large real-world observational cohort study, GLP-1RA use in asymptomatic ICAS patients was associated with lower rates of ischemic stroke (4.40% vs. 6.10%), all-cause mortality (3.40% vs. 9.40%), and the composite endpoint of ischemic stroke or all-cause mortality (7.50% vs. 15.00%) compared to those who did not receive GLP-1RA. Because treatment assignment was nonrandomized and residual confounding remains likely despite PSM, these findings should be interpreted as an association rather than evidence of a definitive protective or causal treatment effect.

### 4.2. Findings in Context of Prior Literature

Prior literature suggests that asymptomatic ICAS carries a comparatively benign short-term prognosis, with reported annual ischemic stroke risks of ~0.6–2.8%, varying by cohort and stenosis severity; for example, an MCA stenosis/occlusion cohort reported ~2.8% annual stroke risk in asymptomatic individuals (vs. ~12.5% in symptomatic disease) [10], while the Northern Manhattan Study reported 12-month event rates of 1.5% ischemic stroke and 6.3% all-cause death among community dwellers with ≥70% stenosis/occlusion [3]. Against this background, our real-world cohort demonstrated slightly higher absolute event proportions (ischemic stroke 6.1% and mortality 9.4% in non–GLP-1RA users, with lower corresponding rates in GLP-1RA users: 4.4% and 3.4%, respectively), which is plausibly consistent with a higher-risk clinical population and longer follow-up than community-based 12-month estimates. While these absolute differences may appear clinically meaningful, particularly in a high-risk cardiometabolic population, they should not be interpreted as treatment effects or directly compared with randomized prophylactic strategies. For asymptomatic ICAS, current management is centered on global vascular risk-factor optimization rather than established ICAS-specific preventive therapy.

Nonetheless, GLP-1RA use within 6 months of diagnosis was associated with lower hazards of ischemic stroke (HR 0.70, 95% CI 0.52–0.95; *p* = 0.044) and all-cause mortality (HR 0.35, 95% CI 0.26–0.47; *p* < 0.001), with a parallel reduction in the composite endpoint, supporting a potential protective effect of GLP-1RA therapy in asymptomatic ICAS, particularly in patients with substantial vascular risk profiles. Given heterogeneity in case ascertainment and outcome capture across datasets, our results are best interpreted as a comparative effectiveness signal in patients with ICAS (lower HRs in GLP-1RA users) rather than as population incidence estimates for asymptomatic ICAS.

The magnitude of the observed mortality association should be interpreted cautiously. Although GLP-1RAs have been associated with cardiovascular benefit in randomized and observational cardiometabolic cohorts, the 1-year mortality HR (0.35) observed in this study is unlikely to reflect a medication effect alone. Alternative explanations include residual confounding, differential healthcare engagement, selection of patients healthy enough to receive or continue GLP-1RA therapy, confounding by indication, unmeasured socioeconomic factors, and incomplete capture of disease severity.

Current ICAS management guidance from major societies—including the American Academy of Neurology and the European Stroke Organisation (ESO)—has largely been developed around symptomatic ICAS, with comparatively limited direction for asymptomatic disease [11,12]. The ESO specifically does not recommend routine population screening for asymptomatic ICAS; however, when clinically meaningful intracranial stenosis is identified incidentally on imaging performed for other indications, it emphasizes that these individuals remain at elevated risk for future stroke and other major vascular events relative to the general population, warranting aggressive vascular risk–factor optimization and lifestyle modification akin to management of a very high-risk patient [12]. The present findings extend the current prevention framework by providing preliminary real-world evidence that GLP-1RA use, within 6 months of ICAS diagnosis, was associated with lower ischemic stroke (HR 0.70) and all-cause mortality (HR 0.35) rates, with a concordant reduction in the composite outcome. While these observational data do not establish causality, they support the hypothesis that GLP-1RAs may represent a future adjunct to standard risk-factor management in selected patients with asymptomatic ICAS, particularly those with high cardiometabolic risk, and motivate prospective studies to define the patients most likely to benefit and to clarify the incremental value over contemporary guideline-directed prevention.

### 4.3. Potential Biological and Vascular Mechanisms

The observed association between GLP-1RA use and lower ischemic stroke rates may be consistent with broader evidence that these agents reduce major adverse cardiovascular events in high-risk cardiometabolic populations, likely through a combination of vascular and systemic cardiometabolic effects. These effects are also particularly relevant to patients with asymptomatic ICAS. Potential mechanisms should also be considered in the context of the distinct biology of intracranial arteries. Compared with extracranial arteries, intracranial arteries have a predominantly muscular wall structure, lack an external elastic lamina, and have relatively sparse vasa vasorum [17,18]. These features may influence plaque development, inflammatory remodeling, endothelial dysfunction, and vulnerability to luminal compromise, and potentially, the later onset of symptoms as compared to extracranial carotid atherosclerosis [17]. In ICAS, ischemic events may arise through artery-to-artery embolism, perforator or branch occlusive disease, and hypoperfusion in the setting of severe stenosis and inadequate collateralization [19]. Mechanistically, GLP-1RAs have been hypothesized to favor atherosclerotic plaque stabilization and slower lesion progression through improved endothelial function and reduced inflammatory remodeling of the fibrous cap, potentially lowering the likelihood of plaque disruption and downstream thromboembolism [20]. In parallel, GLP-1RAs may exert direct neuroprotective and anti-inflammatory effects, including attenuation of neuroinflammation and oxidative stress and preservation of blood–brain barrier integrity in preclinical stroke models, thereby limiting ischemic injury when vascular events occur [15,21,22]. However, the present EHR-based analysis was not designed to test these mechanisms directly. In particular, residual imbalance in HbA1c and BMI after matching, incomplete capture of cardiometabolic control over time, and unavailable data on medication adherence, dose, and duration limit direct mechanistic inference. Therefore, the present findings should be considered a clinical association rather than evidence of direct vasculoprotective or neuroprotective benefit in asymptomatic ICAS.

### 4.4. Limitations

As with any EHR network-based analysis, our study leverages the scale of a multi-institutional dataset but warrants cautious interpretation. First, the retrospective observational design precludes causal inference and remains vulnerable to residual confounding despite PSM on 50 major characteristics. Despite rigorous PSM there was some residual imbalance in BMI and HbA1c distribution. GLP-1RA group had higher BMI and HbA1c values following PSM, and the database did not capture socioeconomic status, medication adherence, healthcare utilization intensity, frailty, diet, exercise, or longitudinal risk-factor control. These factors may influence both the likelihood of receiving GLP-1RA therapy and the risk of stroke or death. Although the higher post-matching BMI and HbA1c in the GLP-1RA cohort would not be expected to explain lower event rates in that group, residual and unmeasured confounding cannot be excluded. Second, cohort classification also relied on structured diagnostic codes rather than centralized imaging adjudication. We attempted to operationalize asymptomatic ICAS by excluding patients with prior or same-day coded cerebral infarction, intracranial hemorrhage, or transient ischemic attack. However, this approach cannot exclude silent infarcts, undocumented transient symptoms, outside events not captured in the network, or coding inaccuracies. Some patients with symptomatic ICAS may therefore have been misclassified as asymptomatic, which could partly explain the higher observed event rates compared with some community-based asymptomatic ICAS cohorts. Conversely, incomplete outcome coding may have underestimated event rates. These limitations reduce certainty regarding the true risk profile of the cohort and limit absolute generalizability to imaging-confirmed asymptomatic ICAS populations. Third, GLP-1RA exposure was based on recorded medication use within the available EHR data and did not capture dose, treatment duration, titration, adherence, persistence, discontinuation, or therapy modifications during follow-up. This may have introduced exposure misclassification and precluded assessment of dose–response relationships or the independent association of sustained GLP-1RA therapy with outcomes. Fourth, structured EHR data do not provide imaging-level ICAS characterization, including stenosis degree, arterial location, plaque morphology, vessel-wall enhancement, collateral status, hemodynamic compromise, or interval revascularization. These missing variables limit risk stratification, mechanistic inference, and external validity. Lastly, we also analyzed GLP-1RAs as a medication class and did not compare outcomes among individual agents. This is relevant because specific GLP-1RA class representatives may differ in pharmacologic properties, dosing schedules, weight-loss and glycemic effects, cardiovascular outcomes evidence, and prescribing patterns. However, agent-specific comparisons may be limited by smaller subgroup sizes, confounding by indication, temporal prescribing trends, and unavailable data on dose, duration, adherence, and persistence. Collectively, these limitations underscore that the observed associations should be viewed as hypothesis-generating and warrant prospective or registry-based studies with standardized intracranial stenosis phenotyping, adjudicated neurologic outcomes, contemporaneous assessment of guideline-directed medical therapy intensity, and longitudinal medication exposure data analyzed using time-dependent exposure modeling or landmark analysis.

In conclusion, in this PSM-matched real-world cohort of adults with coded asymptomatic ICAS, GLP-1RA use was associated with lower observed 1-year risks of ischemic stroke, all-cause mortality, and the composite outcome. These hypothesis-generating findings warrant prospective validation in larger cohorts with standardized ascertainment of medication exposure, dose, and adherence.

## Figures and Tables

**Table 1:** Study outcomes within 1 year of follow-up.

Outcomes	GLP-1RA	No GLP-1RA	Hazard Ratio [95% CI]	*p*-Value
Ischemic stroke	76 (5.18%)	105 (6.98%)	0.70 [0.52–0.95]	0.044
All-cause mortality	58 (4.13%)	162 (10.52%)	0.35 [0.26–0.47]	<0.001
Composite: ischemic stroke or mortality	129 (8.9%)	260 (16.75%)	0.48 [0.39–0.59]	<0.001

Note: Percentages in GLP-1RA and No GLP-1RA columns represent event probability rates.

## Data Availability

Data are available from the corresponding author upon request, subject to TriNetX data-use restrictions.

## Supplementary Materials

The following supporting information can be downloaded at: https://www.mdpi.com/article/10.3390/neurolint18050098/s1, Table S1: ICD-10, RXNORM and LOINC codes used in this study; Table S2: Patient Characteristics Before and After Propensity Score Matching (PSM).
