# Supplementary material for: Association of Glucagon-like Peptide-1 Receptor Agonist Use with Stroke and Mortality Outcomes in Asymptomatic Intracranial Atherosclerotic Disease: Propensity Score-Matched Real-World Analysis
Source: Neurol Int. 2026 May 21;18(5):98. doi: 10.3390/neurolint18050098 (PMC13210162; doi:10.3390/neurolint18050098)
Supplement: Supplementary file 1 [file neurolint-18-00098-s001.zip › neurolint-4280064-supplementary.pdf]

Table S1: ICD-10, RXNORM and LOINC codes used in this study.

Characteristic	Codes (ICD-10 / RXNORM / Other)
**Inclusion**	
ICAD	I67.2
**Exposure**	
Semaglutide	RXNORM: 1991302
Dulaglutide	RXNORM: 1551291
Liraglutide	RXNORM: 475968
Tirzepatide	RXNORM: 2601723
Lixisenatide	RXNORM: 1440051
**Exclusion (for "asymptomatic status")**	
Prior cerebral infarct	I63
Nontraumatic intracerebral hemorrhage	I61
Other and unspecified nontraumatic intracranial hemorrhage	I62
Transient cerebral ischemic attacks and related syndromes	G45
Vascular syndromes of brain in cerebrovascular diseases	G46
**Propensity Score Matching**	
White	2106-3
Black or African American	2054-5
Female	F
Atrial fibrillation & flutter	I48
Hypertension	I10
Hyperlipidemia	E78.5
Diabetes mellitus	E08-E13
Heart failure	I50
Arterial fibromuscular dysplasia	I77.3
Alcohol related disorders	F10
Diseases of liver	K70-K77
Chronic kidney disease	N18
Coagulation defects & hemorrhagic conditions	D65-D69
Peripheral vascular diseases	I73
Ischemic heart diseases	I20-I25
Headache	R51
Mental & behavioral disorders due to substance use	F10-F19
Smoking	Z87.891
Carotid stenosis	I65
Warfarin	RXNORM: 11289
Rivaroxaban	RXNORM: 1114195
Apixaban	RXNORM: 1364430
Dabigatran etexilate	RXNORM: 1037042

Aspirin	RXNORM: 1191
Evolocumab	RXNORM: 1665684
Alirocumab	RXNORM: 1659152
Inclisiran	RXNORM: 2588243
Atorvastatin	RXNORM: 83367
Rosuvastatin	RXNORM: 301542
Pravastatin	RXNORM: 42463
Simvastatin	RXNORM: 36567
Ezetimibe	RXNORM: 341248
Metformin	RXNORM: 6809
Empagliflozin	RXNORM: 1545653
Dapagliflozin	RXNORM: 1488564
Glipizide	RXNORM: 4821
Sitagliptin	RXNORM: 593411
Glimepiride	RXNORM: 25789
Beta blockers/related (class)	CV100
Diuretics (class)	CV700
Calcium channel blockers (class)	CV200
Angiotensin II inhibitor (class)	CV805
ACE inhibitors (class)	CV800
Ticagrelor	RXNORM: 1116632
Clopidogrel	RXNORM: 32968
LDL cholesterol	LOINC: 9002
Hemoglobin A1c	LOINC: 9037
Body mass index	LOINC: 9083
Systolic blood pressure	LOINC: 9085
**Outcome**	
Ischemic stroke	I63
All-cause mortality	Deceased
Composite outcome: Ischemic stroke or mortality	I63; Deceased

Table S2: Patient Characteristics Before and After Propensity Score Matching (PSM).

	Before PSM				After PSM			
**Characteristics - mean +/- SD, % (n)**	**GLP-1RA (N=1,746)**	**No GLP-1RA (N=71,792)**	**P-value**	**SD**	**GLP-1RA (N=1,728)**	**No GLP-1RA (N=1,728)**	**P-value**	**SD**
**Demographics**								
Age (years)	66.2 +/- 10.2	71.7 +/- 12.0	<0.001	0.491	66.2 +/- 10.2	66.3 +/- 12.4	0.839	0.007
White	65.0% (1,135)	70.5% (50,583)	<0.001	0.117	65.1% (1,125)	64.9% (1,121)	0.887	0.005
Black or African American	22.1% (386)	17.9% (12,879)	<0.001	0.104	22.1% (382)	22.7% (393)	0.654	0.015
Female	55.0% (960)	52.4% (37,597)	0.031	0.052	55.1% (952)	54.9% (948)	0.891	0.005
**Diagnosis**								
Atrial fibrillation and flutter	15.6% (272)	17.3% (12,386)	0.067	0.045	15.6% (269)	17.0% (294)	0.249	0.039
Hypertension	81.6% (1,425)	61.5% (44,157)	<0.001	0.457	81.5% (1,409)	82.2% (1,420)	0.627	0.017

Hyperlipidemia, unspecified	58.3% (1,018)	38.4% (27,544)	<0.001	0.407	58.0% (1,002)	57.9% (1,001)	0.973	0.001
Diabetes mellitus	84.6% (1,477)	28.1% (20,200)	<0.001	1.385	84.4% (1,459)	87.4% (1,510)	0.013	0.085
Heart failure	25.3% (441)	17.6% (12,641)	<0.001	0.187	25.2% (435)	26.0% (449)	0.585	0.019
Chronic kidney disease	30.8% (538)	19.6% (14,072)	<0.001	0.26	30.6% (529)	33.2% (573)	0.108	0.055
Peripheral vascular diseases	12.9% (225)	8.8% (6,283)	<0.001	0.133	12.8% (221)	12.8% (222)	0.959	0.002
Ischemic heart diseases	45.3% (791)	32.8% (23,528)	<0.001	0.259	45.1% (780)	46.6% (806)	0.375	0.03
Smoking	23.4% (409)	18.8% (13,489)	<0.001	0.114	23.3% (403)	24.5% (424)	0.402	0.028
Headache	23.1% (404)	14.9% (10,721)	<0.001	0.210	23.0% (397)	23.0% (398)	0.968	0.001
Mental and behavioral disorders due to psychoactive substance use	17.3% (302)	16.9% (12,146)	0.677	0.01	17.4% (300)	16.2% (280)	0.363	0.031
Coagulation defects, purpura and other hemorrhagic conditions	10.7% (187)	10.1% (7,227)	0.377	0.021	10.6% (184)	11.1% (191)	0.702	0.013

Carotid stenosis	34.3% (599)	27.7% (19,890)	<0.001	0.143	34.0% (588)	33.7% (582)	0.829	0.007
**Medication**								
aspirin	42.4% (740)	26.9% (19,327)	<0.001	0.329	42.2% (730)	43.5% (752)	0.45	0.026
clopidogrel	17.0% (296)	8.7% (6,257)	<0.001	0.248	16.8% (291)	17.4% (301)	0.652	0.015
ticagrelor	1.9% (33)	0.8% (556)	<0.001	0.097	1.9% (32)	1.4% (25)	0.35	0.032
warfarin	2.9% (50)	2.8% (1,982)	0.795	0.006	2.9% (50)	4.0% (69)	0.076	0.06
apixaban	11.1% (193)	7.3% (5,208)	<0.001	0.132	11.0% (190)	12.0% (207)	0.364	0.031
rivaroxaban	4.0% (69)	2.1% (1,530)	<0.001	0.106	3.9% (68)	3.6% (63)	0.656	0.015
dabigatran etexilate	0.6% (10)	0.2% (179)	0.008	0.051	0.6% (10)	0.6% (10)	1	<0.001
atorvastatin	49.0% (856)	25.7% (18,444)	<0.001	0.497	48.8% (843)	51.0% (881)	0.196	0.044
rosuvastatin	20.9% (365)	7.5% (5,385)	<0.001	0.391	20.4% (353)	18.6% (322)	0.183	0.045
pravastatin	4.9% (86)	4.1% (2,915)	0.071	0.042	5.0% (86)	4.8% (83)	0.813	0.008
simvastatin	5.2% (90)	4.4% (3,174)	0.141	0.034	5.2% (90)	5.9% (102)	0.373	0.030
ezetimibe	9.0% (157)	2.6% (1,896)	<0.001	0.274	8.7% (151)	8.6% (148)	0.856	0.006
evolocumab	2.9% (51)	0.4% (268)	<0.001	0.201	2.6% (45)	2.5% (44)	0.914	0.004

alirocumab	0.6% (10)	0.1% (90)	<0.001	0.076	0.6% (10)	0.6% (11)	0.827	0.007
beta blocker	56.1% (979)	35.1% (25,211)	<0.001	0.43	55.8% (965)	56.4% (974)	0.758	0.01
angiotensin converting enzyme inhibitors	29.2% (510)	14.9% (10,717)	<0.001	0.35	29.3% (507)	31.4% (543)	0.183	0.045
angiotensin receptor blockers	36.9% (645)	15.8% (11,373)	<0.001	0.493	36.5% (630)	36.6% (633)	0.916	0.004
calcium channel blockers	41.1% (718)	25.5% (18,323)	<0.001	0.336	40.8% (705)	41.3% (714)	0.756	0.011
diuretics	51.0% (890)	26.9% (19,292)	<0.001	0.51	50.7% (876)	51.6% (891)	0.61	0.017
metformin	41.1% (717)	7.1% (5,133)	<0.001	0.864	40.9% (706)	39.1% (675)	0.282	0.037
empagliflozin	18.7% (327)	1.5% (1,111)	<0.001	0.594	18.0% (311)	15.7% (271)	0.069	0.062
dapagliflozin	7.4% (129)	0.8% (549)	<0.001	0.34	7.1% (123)	7.6% (131)	0.602	0.018
sitagliptin	6.0% (105)	1.4% (977)	<0.001	0.249	6.1% (105)	5.6% (97)	0.562	0.02
glimepiride	5.5% (96)	1.0% (731)	<0.001	0.254	5.4% (94)	5.6% (97)	0.823	0.008
**Laboratory**								
LDL cholesterol, mean +/- SD (lab available)	80.6 +/- 38.0 (1,069)	91.1 +/- 39.2 (22,526)	<0.001	0.272	80.7 +/- 37.8 (1,052)	83.5 +/- 39.5 (1,059)	0.103	0.071

Hemoglobin A1c, mean +/- SD (lab available)	7.6 +/- 1.9 (1,334)	6.4 +/- 1.5 (22,868)	<0.001	0.712	7.6 +/- 1.9 (1,318)	7.2 +/- 1.8 (1,344)	<0.001	0.213
BMI, mean +/- SD (value available)	33.4 +/- 7.2 (1,286)	27.8 +/- 6.4 (48,232)	<0.001	0.815	33.4 +/- 7.2 (1,270)	32.2 +/- 7.0 (1,286)	<0.001	0.167
Systolic blood pressure in mm / Hg, mean +/- SD (value available)	131.0 +/- 19.9 (1,419)	134.2 +/- 24.0 (55,533)	<0.001	0.144	131.0 +/- 19.9 (1,402)	132.1 +/- 25.2 (1,461)	0.226	0.045
